# The evaluation of canal wall up cholesteatoma surgery with the Glasgow Benefit Inventory

**DOI:** 10.1007/s00405-019-05670-8

**Published:** 2019-10-04

**Authors:** Johanna Westerberg, Elina Mäki-Torkko, Henrik Harder

**Affiliations:** 1 Division of Neuro and Inflammation Science, Department of Clinical and Experimental Medicine, Linköping University, Linköping, Sweden; 2 Department of Otorhinolaryngology in Linköping, Anaesthetics, Operations and Specialty Surgery Center, Region Östergötland, Linköping, Sweden; 3 Linnaeus Centre HEAD, Swedish Institute for Disability Research, Linköping University, Linköping, Sweden; 4 Faculty of Medicine and Health, Örebro University, 70182 Örebro, Sweden; 5 ENT Clinic, University Hospital, 582 85 Linköping, Sweden

**Keywords:** Cholesteatoma, Health-related quality of life, Glasgow benefit inventory

## Abstract

**Purpose:**

The aim of the study was to investigate the change in health-related quality of life (HRQoL) after canal wall up cholesteatoma surgery, using the Glasgow Benefit Inventory (GBI).

**Methods:**

Data from a consecutive group of 47 adults scheduled for primary cholesteatoma surgery using canal wall up (CWU) with obliteration, from January 2005 to December 2009, were analysed. Information was extracted from a medical database, and complementary data from patient files and audiograms were collected and recorded retrospectively. The GBI questionnaire was used for the assessment of HRQoL after surgery.

**Results:**

There was no finding of residual or recurrent cholesteatomas in the study group. Hearing was improved at 1 and 3 years postoperatively. No patient suffered a total hearing loss. The overall GBI scores showed an improved HRQoL after surgery. Twenty-nine (85%) patients benefitted from surgery, 1 (3%) had no change, and 4 (12%) expressed deterioration.

**Conclusions:**

Cholesteatoma surgery using CWU with obliteration gives an improved HRQoL for the majority of patients. The GBI questionnaire provides complementary information to hearing and healing results after cholesteatoma surgery.

## Introduction

Cholesteatomas are benign bone destructive expansions of stratified squamous epithelium in the temporal bone, which are classified into congenital or acquired [1]. They are frequently associated with chronic otitis media (COM) but should be considered a separate entity. They are associated with symptoms such as hearing loss, otorrhoea, facial nerve palsy, and vertigo, and if left without treatment, they can pose a risk for potentially lethal complications, such as meningitis and brain abscess [2]. The treatment is an often challenging surgical procedure, with the aim to eradicate the disease, restore hearing, and avoid recurrent cholesteatomas [3]. The measurements of cholesteatoma surgical outcomes are, in general, presented in terms of the disease control rate and hearing results, but reports of, and the interest for, health-related quality of life (HRQoL) assessments are increasing [4]. Amongst HRQoL assessments with the inclusion of cholesteatomas, various questionnaires have been used, such as the chronic ear survey (CES) [5–7], the five-item quality of life survey (COM-5) [8], the Chronic Otitis Media Questionnaire 12 (COMQ-12) [9], the Korean version of the chronic ear survey (K-CES) [10], and the Zurich Chronic Middle Ear Inventory (ZCMEI-21E) [11]. The Glasgow Benefit Inventory (GBI) is a questionnaire designed for post-interventional HRQoL changes in otorhinolaryngological (ORL) procedures. It has the advantage of enabling comparison across different interventions and the ease of being single administered, and it has gained widespread popularity since reported by Robinson et al. [12]. A systematic review of the literature was conducted on the reported use of the GBI, with available data for tonsillectomy, cochlear and middle ear implantation in patients with vestibular schwannoma, and stapes surgery [12]. Besides Robinson et al. [13], according to our knowledge, there is only one previous study reporting cholesteatoma surgical outcomes by means of the GBI [14]. By using the GBI, Maile et al. [14] showed improved HRQoL among 31 Nepali patients after middle ear and/or mastoid surgery to eradicate cholesteatoma. The surgical method used or surgical outcome in terms of healing and hearing results was not reported [14].

The primary aim of the current study was to investigate the patient-reported change in HRQoL after cholesteatoma surgery, using the GBI. Further, the study aimed to explore if the opinions of patients were in agreement with those of surgeons in terms of low residual frequency and generally improved hearing, as a successful cholesteatoma surgical outcome, as described in our earlier study [15].

## Materials and methods

### Setting and procedure

Patients aged 15 years or older who had primary surgery for congenital or acquired cholesteatoma at a tertiary-stage hospital, Linköping University Hospital, and a secondary-stage hospital, Vrinnevi Hospital, Norrköping, between January 2005 and December 2009 were included. Exclusion criteria were isolated atticoantrotomies, isolated myringo-ossiculoplasties, and canal wall down (CWD) procedures. Further, exclusion criteria were children under the age of 15 years at the time of surgery and patients who were not able to answer the questionnaire because of mental disorders, dementia, or poor knowledge of the Swedish language, needing an interpreter. The GBI questionnaire, information about the study, and a prepaid self-addressed envelope were sent to all patients included in the study (*n* = 47) in 2012. A written informed consent was received from the participants or the guardian if the patient was under the age of 18 years. Non-respondents were contacted twice to be reminded of the study. Cholesteatoma surgery using canal wall up (CWU) with obliteration was performed as described in our previous study [15].

### GBI questionnaire

The GBI is an 18-item questionnaire for measuring changes in health-related benefits post-ORL interventions. The questions are answered using a 5-point Likert scale, ranging from a large deterioration (1 point) to a large improvement (5 points) in health status. To avoid response bias, half of the questions are graded from a large deterioration to a large improvement and half in the opposite order. The GBI contains a total score (18 questions) and three sub scores: general benefit (12 questions), social support (3 questions), and physical benefit (3 questions). When calculating the scores, the mean score for each group is subtracted by 3 and multiplied by 50. The worst possible change becomes − 100, and the best possible change becomes + 100 [13].

### Audiometry

Audiograms were accessed from patient files. The pure tone average (PTA) was defined as the mean value of hearing thresholds at the frequencies 0.5, 1.0, 2.0, and 3.0 kHz, as proposed by the British Society of Audiology [16]. A hearing threshold of 30 dB PTA was used as the level for socially adequate hearing [17]. Air conduction thresholds exceeding the maximum output level of clinical audiometers were registered as 130 dB HL to enable the assessment of possible postoperative deterioration and avoid unnecessary dropouts [16]. Bone conduction thresholds exceeding the maximum output level of clinical audiometers were registered as missing. The audiometry was performed with clinical audiometers (Madsen OB822, Otometrics, Denmark), at ear, nose and throat clinics in Linköping and Norrköping. The audiometers were regularly calibrated according to international standards [18]. Audiograms selected as preoperative were the ones performed closest to surgery. One-year postoperative hearing results were based on audiograms obtained closest to 1 year after surgery, with the earliest accepted time being 9 months postoperatively. Three-year postoperative hearing results were based on audiograms obtained closest to 3 years after surgery, with the earliest accepted time being 1.5 years postoperatively. The gain was calculated as preoperative–postoperative PTA.

### Statistical analysis

Statistical analyses were performed using IBM^®^ SPSS^®^ (version 23), Microsoft Excel, and Medlog^®^ database program. Linear regression analysis was used to determine the association between GBI score and age, gender, years since surgery, 1-year postoperative gain, PTA, and mean PTA ≤ 30 dB. Audiometric results and GBI scores are reported as mean ± standard deviation (SD).

## Results

Seventy-six patients were identified in the consecutive group of primary cholesteatoma surgeries within the time frame of the study. Twenty-five children under the age of 15 years were excluded from this group. There was a further exclusion of four adults: two of them because of their poor knowledge of the Swedish language, one because of intellectual disability, and one because of dementia. In the final study group, there were 47 patients, of which 34 (72%) returned a filled-in questionnaire. Demographic data and preoperative findings for the study group as well as for the non-respondents are provided in Table 1. The oldest patient was 76 years, whereas the youngest was 15 years; the mean age was 44 years at the time of surgery. Four patients were between 15 and 17 years old. Ten (29%) patients had an ongoing infection preoperatively in the respondent group. At the clinical control, all operated ears were healed and water resistant at 1 year postoperatively. Diffusion weighted MRI was not performed postoperatively on the study group.

**Table 1:** Description of patient characteristics and peroperative findings in the study group (*n* = 34)

Characteristics	Respondents (*n* = 34) *n* (%)	Non-respondents (*n* = 13) *n* (%)
Diseased ear		
Right	19 (56)	4 (31)
Left	15 (44)	9 (69)
Gender		
Female	18 (53)	8 (62)
Male	16 (47)	5 (38)
Hospital where surgery was performed		
Linköping	20 (59)	7 (54)
Norrköping	14 (41)	6 (46)
Surgeon		
Senior	26 (76)	11 (85)
Junior (working under the supervision of a senior surgeon)	8 (24)	2 (15)
Peroperative findings		
Origin of cholesteatoma		
Attic	24 (71)	6 (46)
Tensa	7 (21)	5 (38)
Combined attic and tensa	2 (6)	1 (8)
Intratympanic/congenital	1 (3)	1 (8)
Ossicular chain		
PORP	26 (76)	10 (77)
TORP	4 (12)	2 (15)
No ossicular reconstruction	4^a^ (12)	1^a^ (8)
Labyrinthine fistula	2 (6)	1 (8)
Infection	10 (29)	5 (38)

*PORP* partial ossicular replacement prosthesis, *TORP* total ossicular replacement prosthesis

^a^In one patient, ossicular reconstruction was not possible because of a facial nerve dehiscence

### Surgery

In all patients, a CWU procedure with obliteration was performed. There were no findings of residual or recurrent cholesteatomas in the study group. Partial ossicular replacement prosthesis (PORP) and total ossicular replacement prosthesis (TORP) were used for ossicular reconstruction in 26 and 4 patients, respectively. The autologous incus or cortical bone was chosen for ossicular reconstruction. Fascia or perichondrium was used as a graft material for the tympanic membrane. Ossicular reconstruction was not performed in four patients. In one of these four patients, an ossicular chain defect was detected, but reconstruction was not possible because of a facial nerve dehiscence. Surgery was performed by a senior surgeon on 26 (76%) of the patients and by a junior surgeon on the rest of the patients under the supervision of a senior surgeon (see Table 1).

### Hearing results

There was an overall hearing improvement in the study group (*n* = 34) at 1 and 3 years postoperatively. Audiograms were possible to achieve in 33 patients at 1-year and in 27 patients at 3-year postoperative follow up. The mean PTA for air conduction was 47 dB (SD 20) preoperatively, and 29 dB (SD 18) at 1 year and 33 dB (SD 21) at 3 years postoperatively. In the PORP subgroup (*n* = 26), 1- and 3-year follow up audiograms were achieved in 25 and 21 patients, respectively, with a mean PTA for air conduction being 45 dB (SD 19) preoperatively, 25 dB (SD 10) at 1 year and 28 dB (SD 13) at 3 years postoperatively. A follow up with audiogram in the TORP (*n* = 4) group as well as in the group of “no reconstruction of ossicular chain” (*n* = 4) was achieved in four patients at the one year postoperative control, and in three patients at the three year postoperative control. In the TORP subgroup, the mean PTA for air conduction was 61 dB (SD 20) preoperatively, and 52 dB (SD 33) at 1 year and 39 dB (SD 16) at 3 years postoperatively. In the group without ossicular reconstruction, the mean PTA for air conduction was 42 dB (SD 25) preoperatively, and 35 dB (SD 19) at 1 year and 51 dB (SD 34) at 3 years postoperatively. Gain at 1 year postoperatively was overall improved. The highest mean gain, 20 dB (SD 16), was seen in the PORP subgroup. The proportion of patients within PTA ≤ 30 dB increased from 9 (26%) to 20 (61%) at 1 year postoperatively and to 15 (60%) at 3 years postoperatively. An interaural difference of ≤ 15 dB was obtained in 18 (58%) of the patients at 1 year postoperatively, compared to 7 (21%) of the patients preoperatively. As described above, the restoration of hearing was not possible in one of the patients. The same patient suffered from deteriorated hearing at 2 years postoperatively, without any known reason. In one patient, an ossiculoplasty revision was performed after the 1-year and before the 3-year postoperative audiograms were recorded. There were no cases of total hearing loss after surgery. Four patients presented a negative overall GBI score. Of these, the mean PTA for air conduction of ≤ 30 dB was achieved in two of the patients, whereas two of them had a mean of > 30 dB at 1 and 3 years postoperatively. The interaural PTA air conduction difference was between 19 and 34 dB in four patients with a negative GBI score.

### Hearing results for non-respondents

In the non-respondent group (*n* = 13), the mean PTA for air conduction was 39 dB (SD 22) preoperatively, compared to 38 dB (SD 26; *n* = 13) and 42 dB (SD 27; *n* = 9) at 1 and 3 years postoperatively. The mean gain was 3 dB (SD 13; *n* = 11) at 1 year postoperatively and 2 dB (SD 14; *n* = 9) at 3 years postoperatively. The proportion of patients with the mean PTA for air conduction of ≤ 30 dB was 6 (46%; *n* = 13) preoperatively, compared to 7 (58%; *n* = 12) and 4 (44%; *n* = 9) at 1 and 3 years postoperatively. No cases of total hearing loss after surgery were seen.

### GBI

The means and SDs of GBI scores were reported. The overall GBI score was 21 (SD 22). In the group of 4 patients under the age of 18, the total GBI score was 39 (SD 26). In the PORP, TORP, and no ossicular reconstruction subgroups, the total scores were 24 (SD 23), 19 (SD 8), and 8 (SD 11), respectively. The general, social, and physical health subscale scores for the total study group were 26 (SD 27), 10 (SD 21), and 13 (SD 24), respectively, as provided in Table 2. Twenty-nine (85%) of the patients benefitted from surgery, one (3%) patient had no change, and four (12%) expressed deterioration after surgery (see Table 3). One patient scored between 1 and 2 in question 1, and we chose to use the lower score for calculations. There was no effect on the GBI score of age, gender, years since surgery or 1-year postoperative gain, PTA, or mean PTA ≤ 30 dB, as mentioned in Table 4.

**Table 2:** Mean GBI (total and subscale) scores for 34 respondents

Study group	*n*	GBI Mean (SD)			
		Total score	General score	Social support	Physical support
All	34	21 (22)	26 (27)	10 (21)	13 (24)
Subgroups					
No ossicular reconstruction	4	8 (11)	10 (12)	0 (0)	− 2 (29)
PORP	26	24 (23)	28 (29)	12 (23)	17 (24)
TORP	4	19 (8)	28 (12)	4 (7)	4 (7)

*GBI* Glasgow Benefit Inventory, *SD* standard deviation, *PORP* partial ossicular replacement prosthesis, *TORP* total ossicular replacement prosthesis

**Table 3:** GBI (total and subscale) scores with the percentage of improved, unchanged, and deteriorated patients after cholesteatoma surgery (*n* = 34)

GBI score	Median (range)	GBI change *n* (%)		
		Improved	Unchanged	Deteriorated
Total	19 (− 28 to 75)	29 (85)	1 (3)	4 (12)
General	26 (− 42 to 75)	28 (82)	2 (6)	4 (12)
Social support	0 (0 to 83)	9 (26)	25 (74)	0
Physical health	0 (− 17 to 75)	17 (50)	14 (41)	3(9)

*GBI* Glasgow Benefit Inventory

**Table 4:** Separate regressions of one of several explanatory variables and overall GBI score

Explanatory variable	Slope (±SE)	*t*	*p*	*R*^2^
Age	− 0.2 (0.2)	− 0.9	0.36	0.03
Gender	− 10.1 (7.4)	− 1.3	0.19	0.05
Gain	0.35 (0.24)	1.48	0.15	0.06
Years since surgery	0.70 (2.98)	0.23	0.82	0.00
*1-year postoperative*				
Mean PTA	− 0.28 (0.21)	− 1.32	0.198	0.05
PTA ≤ 30 dB	− 0.28 (0.21)	− 1.32	0.2	0.02
Interaural difference	− 0.42 (0.28)	− 1.53	0.14	0.07

Coefficients (slope ± standard error) and test results (*t* and *p* value, and amount of variation explained, *R*^2^) from separate regressions with the overall GBI score as dependent variable

*GBI* Glasgow Benefit Inventory, *PTA* pure tone average, *SE* standard error

## Discussion

A majority of patients, 29 (85%) of 34, presented a positive total GBI score after cholesteatoma surgery. The scores were also positive in the general, social support, and physical support subgroups. A minor group of 4 (12%) patients declared deterioration after surgery. Hearing was improved overall at 1 and 3 years postoperatively, without residual or recurrent cholesteatomas.

Cholesteatoma is a disease with a heterogenic appearance, and the variation amongst symptoms can be expected to contribute to the differences in experienced HRQoL. In a study by Vlastos et al. [8], a group of 19 children showed poor results on responsiveness to change with the COM-5 after cholesteatoma surgery. According to the authors, the limited symptoms and limited functional problems of some patients preoperatively could explain why surgery did not improve HRQoL, despite a dramatically changed ear status postoperatively. Although Vlastos et al. [8] have reported results from a paediatric population (aged 4–14 years), we find their conclusion to be applicable to the present study. The heterogenicity in symptoms in cholesteatoma ears from a lot of symptoms to no symptoms at all preoperatively could explain the spread of post-surgical benefit in HRQoL. Nadol et al. [7] saw a positive impact on the patients’ health after surgery for COM, using the disease-specific outcome survey, the CES. The improvement was according to total score, moderate amongst the cholesteatoma patients. A very little improvement was seen in the subscale of activity restriction, which covers the impact of COM on the patient´s daily life. The authors suggest that water restrictions during the mastoid healing process have influenced the lower scores of the cholesteatoma group. In our study group, all ears were water resistant at one year after surgery, and we could not relate the GBI scores to drainage or non-waterproof ears.

The GBI scores are further not necessarily in line with the healing and hearing results according to our results. It is more difficult to explain that there is no correlation between GBI scores and amelioration of hearing in the present study. In a study by Choi et al. [10], patients referred for ear surgery (COM with or without cholesteatoma) were assessed with the K-CES. In their study, post-surgical results showed an association between a limited HRQoL improvement and postoperative (not specified) complications, hearing loss as a chief complaint, worse postoperative air conduction thresholds, higher level of education, CWD mastoidectomy, or diabetes mellitus; the latter was possibly associated with impaired wound healing [10]. In the present study, except for postoperative air conduction thresholds, we did not investigate the parameters as in the study by Choi et al. [10]. Postoperative hearing was in general improved at 1 and 3 years postoperatively, and we did not detect any effect on the overall GBI scores of 1-year mean PTA.

Maile et al. [14] investigated the GBI scores in patients from a poorly developed area of Nepal, showing excellent postoperative benefit of cholesteatoma surgery. The authors concluded that the patient responses might be affected by a desire to please health care professionals or influence future care. Furthermore, they also identified a potential source of bias, as there might be an incentive for the local “community ear assistants”, who collected data to demonstrate that their care had a positive impact on the HRQoL of patients. In comparison to the study by Maile et al. [14], the present study shows GBI scores that are much lower. These results are more in line with the findings by Robinson et al. [13], in which the total GBI scores were reported from − 2 to + 17 after surgery for eradicating ear activity or ear discharge [13]. In the present study, a preoperative middle ear infection was only present in ten (29%) of the patients and no patient suffered from ear discharge at 1-year postoperative follow-up.

The expectations of patients with regard to cholesteatoma surgical outcomes are crucial for their postoperative satisfaction. Communication with patients prior to surgery is of great importance for the best possible agreement and realistic expectations. There is often a challenging surgical situation as radicality might be in conflict with functionality in the short perspective. As mentioned above, cholesteatoma presents with only few symptoms or none at all in some patients. This must be taken into consideration when proposing surgery. Good postoperative compliance is necessary for a good long-time result. One of the challenges is to motivate patients to perform the Valsalva manoeuvre when needed and to avoid sniffing to prevent the recurrent cholesteatoma and the failure of ossicular reconstruction [19].

Robinson et al. [13], validated the GBI questionnaire for the sensitivity to change in health status for different groups of patients, post ORL interventions. Pediatric population was not excluded, but the group of tonsillectomy, with a mean age of 10 years were excluded from the factor analysis as data were obtained by proxy (parents were asked to complete the GBI on behalf of the child). In the present study we chose to exclude children under the age of 15 years. The four patients between 15 and 17 years old, had a total GBI score of 39, not altering the results for the total study group, namely an improved HRQoL after cholesteatoma surgery.

### Response rate and participants

A poor response rate is not seldom the case in retrospective questionnaire studies. There is, thus, a risk that the participants in the study would only represent a smaller part of the entirety. From the consecutive group of patients with performed cholesteatoma surgery using CWU in the present study, there was an equal participation of males 16 (47%) and females 18 (53%) and also the spread of ages was equal (15–76 years). As we routinely have a 5-year follow-up for adults and a 10-year follow-up for children (<18 years) after cholesteatoma surgery, the majority of patients in the study group were still regularly offered clinical check-ups. This fact might have been an incitement to participation for some patients. The response rate was 72%, which is the same percentage as presented in the study by Robinson et al. [13], for the ear surgery group aiming at eradicating ear activity.

### Time since surgery

Robinson et al. [13] studied 138 patients for a maximum of 6 years after ear surgery. Despite the limitation of a retrospective questionnaire as the memories of patients of surgery and its affects would fade with time, it seems that it did not affect the overall score [13]. This was in line with our data, where the longest time from surgery to answering the questionnaire was 7 years. The results of the GBI questionnaire give information about the present status of the patients and have to be interpreted in relation to the “time factor”. Maile et al. [14] had a follow-up at “the postoperative clinical appointment” and suggested a later GBI follow-up to their study group to quantify long-term changes in HRQoL following surgery [14].

### Hearing and expected GBI score

Smyth and Patterson [20] identified higher satisfaction among patients after myringo-ossiculoplasties in case of smaller interaural hearing differences, greater gains, and better postoperative hearing thresholds. According to the Belfast rule of thumb, patients would not consider their hearing to be improved unless the hearing threshold is ≤ 30 dB or within 15 dB of the contralateral ear [12]. Nadol et al. [7] saw a strong correlation between the preoperative and postoperative hearing results and the CES scores. We did not see a significant effect on the overall GBI score of postoperative hearing data (Table 4), but four patients with a negative total GBI score had a greater interaural difference than that of the mean of the total group. A correlation between hearing amelioration and GBI results could have been expected. The modest GBI scores, despite a low frequency of recurrence and residual cholesteatomas, a lack of discharge problems and only one ossicular revision in the study group, could possibly partly be explained by the fact that the symptoms preoperatively were scanty.

### Negative scores

Hendry et al. [21] recommended the reporting of GBI scores of the percentage of patients for whom there was no or negative benefit with surgery. As seen above, four (12%) of the patients had a negative total score. The first patient, with − 28 as the total GBI score, had acute surgery for labyrinthitis and a labyrinthine fistula caused by cholesteatoma. PTA air conduction was 44 dB at 1 year postoperatively, and a subsequent sudden hearing loss occurred at 2.5 years postoperatively. MRI and CT scans were performed with no detected residual or recurrent disease.

The second patient with a deteriorated GBI score (− 8) had an attic cholesteatoma, a history of Eustachian tube dysfunction, and ear problems since childhood. There were no surgical complications. At 1-year postoperative control, PTA air conduction was 20 dB with a gain of 15 dB, compared to preoperative hearing. The questionnaire was filled in 4 years postoperatively. In this case, it is difficult to explain why the patient’s GBI score was low, but it might mirror the difficult task to convey realistic expectations of surgery.

The third patient with a deteriorated GBI score (− 6) had an attic cholesteatoma. Because of a difficult anaesthetic situation, the surgery had to be cancelled and postponed to a few days later for awake intubation. At 1 year postoperatively, the patient had a conductive hearing loss with a mean PTA for air conduction of 37 dB because of the fixation of ossicular reconstruction and retraction problems contributing to a difficult hearing situation.

The fourth patient with a deteriorated GBI score (− 5) also with an attic cholesteatoma showed a mean PTA for air conduction of 19 dB at 1 year postoperatively. By the time of filling in the questionnaire, tinnitus had newly developed in the contralateral ear. An MRI was performed, but it did not show any detectable pathology.

It was not possible to address the negative GBI scores to a specific postoperative parameter in this study, but hearing problems and possibly interaural difference in hearing thresholds could partly be responsible for the deterioration. The number of labyrinthine fistulas peroperative were too small in this study group for drawing further conclusions. One of the patients with labyrinthine fistula showed negative GBI scores, one had positive GBI scores, and the third unfortunately did not answer the questionnaire.

### Limits of the study

The Swedish translated version of the GBI questionnaire is currently being validated by a Swedish research group, but the conclusion is not yet published. However, the GBI questionnaire is used in the clinic by several members of the Swedish Association for Ear Surgery.

The sample size is small, and in the study group, there was only one revision surgery and no residual or recurrent disease, which makes generalization difficult. Data from the current 34 patients are in line with the group of 230 patients from our earlier study [15], showing a low residual and recurrent rate. In the study by Maile et al. [14], 31 patients with cholesteatoma were included, which is a sample size similar to ours. As there is a lack of publications in the field of HRQoL and GBI for patients with cholesteatoma, comparison with other studies is limited. In the subgroups of “no reconstruction of ossicular chain”, and the TORP group only contains four patients, and at the 3 year follow up only three patients present hearing data. This could explain the ameliorated hearing between one and 3 year postoperative control.

There is always a risk of positive bias after interventions, which is not unique to our study, as patients tend to appreciate the surgical attention and caretaking [22]. To dampen this effect, an impartial questionnaire administration could possibly be an advantage.

Another general problem with questionnaires is exclusion on the basis of language skills. Patients with poor knowledge of the Swedish language were excluded from the study, which has to be considered when interpreting these data. However, the number of non-Swedish-speaking patients excluded from the study was low in this study group.

### Strengths and advantages of the study

The study group is part of the cohort earlier described [15]. Therefore, these data are useful as a complement to the previously reported hearing data and surgical outcome. The GBI is a non-disease-specific tool for the measurement of HRQoL in contrast to, for example, the CES, but to its advantage, it is possible to make comparisons between different interventions [23].

## Conclusions

Cholesteatoma surgery using CWU with obliteration gives an increased quality of life for a majority of patients. The GBI is a complement to hearing and healing results after cholesteatoma surgery to provide useful information to the caregiver. It can be a helpful tool to identify some of the postoperative challenges in cholesteatoma surgery.

Low and even negative GBI scores were detected after cholesteatoma surgery, although no residual or recurrent cholesteatomas were seen, and some of these patients had ameliorated hearing, which mirrors the complexity of cholesteatoma surgery and reveals difficulty in using the GBI in this group of patients.

The GBI scores most probably provide information about the expectations of patients, as well as the objective hearing and healing results of surgery. Further studies are suggested for an understanding of the complexity of cholesteatoma surgical outcomes in comparison to the opinions of patients and to possibly detect the key factors associated with HRQoL.
